# Shifted Balance Between Ventral Striatal Prodynorphin and Proenkephalin Biases Development of Cocaine Place Avoidance

**DOI:** 10.1111/adb.70055

**Published:** 2025-07-06

**Authors:** Amélia Nicot, Pavankumar Yecham, Ilana Serin, David J. Barker, Lauren K. Dobbs

**Affiliations:** ^1^ Department of Neuroscience, Waggoner Center for Alcohol and Addiction Research The University of Texas at Austin Austin Texas USA; ^2^ Department of Psychology Brain Health Institute, Rutgers, The State University of New Jersey Piscataway New Jersey USA; ^3^ Department of Neurology Dell Medical School, The University of Texas at Austin Austin Texas USA

**Keywords:** dynorphin, enkephalin, individual differences, negative affect, place aversion, striatum, trace conditioning

## Abstract

Evidence from human self‐report and rodent models indicate that cocaine can induce a negative affective state marked by panic and anxiety, which may reduce future cocaine use or promote co‐use with opiates. Dynorphin‐mediated signalling within the striatum is associated with negative affect following cocaine withdrawal and stress‐induced cocaine seeking. Here, we used a trace conditioning procedure to first establish the optimum parameters to capture this transient cocaine negative affective state in wild‐type mice, and then we investigated striatal opioid peptides as a substrate mediating cocaine conditioned place avoidance (CPA). Previous reports indicate that trace conditioning, where drug administration occurs after removal from the conditioning chamber, results in CPA to ethanol, nicotine and amphetamine. We tested different cocaine doses, conditioning session lengths and apparatus types to determine which combination yields the best cocaine CPA. Cocaine CPA was moderately larger at the highest cocaine dose (25 mg/kg), but this did not generalize across apparatus types and the effect was transient; thus, data were collapsed across all parameters. Cocaine conditioning scores were variable but also became more polarized across conditioning, with approximately equal proportions developing preference and avoidance. We then correlated cocaine CPA with striatal gene expression levels of the opioid peptides enkephalin (*Penk*) and dynorphin (*Pdyn*) using qPCR. Cocaine CPA was correlated with low *Pdyn* levels and a low *Pdyn*:*Penk* ratio in the ventral, but not dorsal, striatum. Consistent with this, mice with higher striatal *Pdyn* relative to *Penk* were more resistant to developing cocaine CPA compared with littermate controls. This approach revealed a subset of subjects sensitive to the aversive state immediately following cocaine administration. Our findings suggest that striatal dynorphin has opposing roles in mediating the aversion associated with acute cocaine intoxication versus cocaine withdrawal.

## Graphical abstract

We examined the optimum parameters for producing conditioned place avoidance (CPA) to the acutely aversive effects of cocaine treatment. We discovered that individual differences, rather than cocaine dose, apparatus type, stimulus modality or conditioning time, were important for expression of cocaine CPA. We further discovered that CPA was associated with low *Pdyn*:*Penk* mRNA levels in the ventral striatum of wild‐type mice and causally demonstrated that transgenic mice with pre‐existing higher striatal *Pdyn*:*Penk* expression levels were more resistant to develop CPA. Created in BioRender. Dobbs, L. (2025) https://BioRender.com/b2agzur.

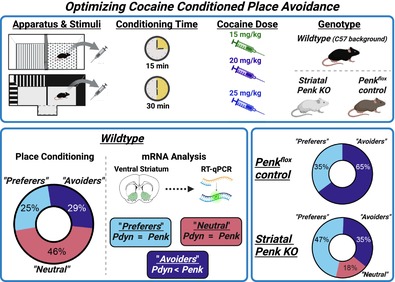

## 1 Introduction

Recent reports indicate the rate of cocaine use in the United States is rising [1]; however, there are no FDA‐approved pharmacotherapies and existing off‐label medications have limited treatment success [2]. Compulsive seeking and taking are hallmarks of cocaine use disorder, and these behaviours are driven by a combination of positive and negative reinforcement mechanisms. Although cocaine‐induced feelings of euphoria contribute to positive reinforcement, withdrawal from cocaine can provoke an aversive affective state that drives continued cocaine seeking and taking [3, 4, 5, 6, 7]. In contrast, acute cocaine intoxication can also induce an aversive affective state, which manifests as a sympathomimetic response, including feelings of paranoia and panic attacks [8, 9, 10]. Epidemiological data indicate that cocaine use increases the risk of experiencing panic attacks [8]. Although this may only occur in a subset of individuals, these negative experiences often lead to decreased cocaine use or stopping altogether [10]. Taken together, it appears that acute cocaine intoxication can induce bivalent affective states associated with reward and aversion, which generate motivational drives to either seek or avoid cocaine. Accordingly, developing a better understanding of the neurobiology underlying the balance between the rewarding and aversive aspects of acute cocaine intoxication will provide insight into vulnerability and protective factors associated with risk to develop cocaine use disorder and may reveal new targets that can be leveraged for treatment.

Although numerous rodent models exist of the positive reinforcing and rewarding aspects of cocaine, including conditioned place preference (CPP) and operant self‐administration, there is limited information on how to best model the aversion associated with acute cocaine use. Acute cocaine has been reported to induce thigmotaxis and decreased time spent in the open arms of an elevated plus maze, suggesting that it can induce an aversive state in rodents [11, 12, 13]. In a drug self‐administration runway procedure, rats develop an approach‐avoidance conflict to a cocaine‐associated goal box over progressive training trials ([14, p. 199]; [6, 15, 16, 17, 18]). In this procedure, subjects traverse a straight‐alley runway to receive intravenous cocaine (0.75–3.75 mg/kg) when they reach the goal box and then remain in the goal box for 5 min. During this time, the animal associates the goal box with cocaine's rewarding and aversive properties. Thus, although subjects are in an undrugged state while traversing the runway, over successive training trials, subjects condition an association between the goal box and the drugged state, which results in the expression of approach‐avoidance behaviour during locomotion to the goal box on subsequent trials. However, with this procedure, it is difficult to parse whether the avoidance that develops is the result of cocaine intoxication‐associated or cocaine withdrawal‐associated aversion.

Place conditioning is a commonly used paradigm to model the motivational effects of drugs in experimental animals (see reviews by [19, 20]). The rewarding and aversive effects of drugs (the unconditioned stimulus, US) are inferred based on how much time the subject spends on a drug‐paired floor (the conditioned stimulus, CS). For instance, a simultaneous pairing of the CS and US typically leads to the development of preference for a wide range of drugs of abuse, including cocaine, ethanol, morphine, amphetamine and methamphetamine [21, 22, 23, 24]. Specifically, when cocaine is administered 0 min (i.e., simultaneous pairing) or 5 min before being placed in the conditioning chamber, a CPP develops, likely as a consequence of pairing the US with ‘peak or near peak brain levels’ of cocaine [4]. Conversely, when cocaine is administered 15 min before being placed in the conditioning apparatus, a conditioned place avoidance (CPA) develops. Based on the pharmacokinetics of cocaine, this is thought to arise from pairing the aversive aspect of rapidly falling brain levels of cocaine with the conditioning chamber [4]. In addition to CPA developing as a consequence of drug withdrawal, several reports indicate that the acutely intoxicating effects of drugs of abuse, including ethanol, nicotine and amphetamine, can be aversive [25, 26, 27]. In this conditioning procedure, exposure to the CS precedes the US [28]. Rodents are confined to one side of a conditioning apparatus for a specific length of time and then removed, immediately administered a drug injection and returned to their home cage. This CS–US pairing order is referred to as ‘trace’, or ‘delay’, depending on whether there is a temporal gap between CS and US presentation (trace) or if CS–US presentation overlaps to some extent (delay). This order of CS–US pairing is thought to pair a transient, drug‐induced aversive affective state with the preceding context. In support of this, trace conditioning with vehicle does not produce a CPA [26, 27], and CPA does not develop when increasing temporal gaps are introduced between the context exposure and ethanol administration [25]. This suggests that the drug imbues the preceding context with motivational significance. Further, the doses of ethanol, nicotine and amphetamine that produce CPA in a trace conditioning procedure are the same as doses that condition place preference when the CS and US are simultaneously paired, suggesting that these different conditioning modalities capture distinct, bivalent affective states of these drugs.

Compared with our understanding of the neurobiological mechanisms underlying cocaine reward, the mechanisms driving cocaine aversion remain relatively uncharacterized. Moreover, much of the knowledge regarding cocaine aversion has focused on mechanisms associated with reinstatement of cocaine seeking following short (4 days) or protracted (up to 30 days) cocaine withdrawal, as opposed to the acutely aversive state induced immediately after cocaine administration. In particular, there is substantial evidence for a role of kappa opioid receptor (KOR) activation by the opioid peptide dynorphin in the aversion associated with repeated cocaine treatment and cocaine withdrawal. Expression levels of prodynorphin (*Pdyn*), the gene that encodes dynorphin, increase in the dorsal striatum following repeated, but not acute, cocaine treatment [29]. This increased dynorphin tone appears to be an adaptive response to repeated activation of D1 receptor–containing MSNs (for a review, see [30]). Increased dynorphin‐mediated KOR signalling also appears to act as a negative reinforcer and increase cocaine seeking. Selective KOR agonists reinstate cocaine seeking after 1 month of cocaine withdrawal during extinction training in an operant procedure [31]. Further, stress‐induced release of dynorphin reinstates cocaine seeking after extinction, and this has been reported for a wide range of cocaine withdrawal times (4 days–1 month) [32, 33, 34]. Dynorphin‐mediated KOR signalling in distinct regions of the ventral striatum is also acutely aversive by itself [35, 36], and activation of KORs in the nucleus accumbens core or ventral tegmental area potentiates nicotine conditioned taste aversion, suggesting that KORs may exacerbate the acutely aversive aspect of nicotine [37]. In contrast, the activity of mu‐opioid receptors (MOR) in the ventral striatum is critical for conditioning the rewarding properties of cocaine. Systemic and intraventral striatum administration of MOR antagonists blocks the acquisition and expression of cocaine place preference [23, 38, 39]. Additionally, enhancing the level of enkephalin, an opioid peptide with a high affinity for MORs and delta‐opioid receptors (DOR), within the ventral striatum facilitates the acquisition of cocaine place preference [23]. Taken together, these data suggest that within the ventral striatum, dynorphin‐mediated activation of KORs may facilitate withdrawal‐associated and perhaps even intoxication‐associated cocaine aversion, whereas enkephalin‐mediated activation of MORs supports cocaine's acutely rewarding state.

Despite evidence that cocaine possesses aversive qualities independent of withdrawal, to date, there are no published reports of cocaine CPA for the acutely aversive effects of cocaine. Thus, we predicted that these could be modelled and readily captured using a trace conditioning procedure, which has been used to capture the acutely aversive states of several other abused drugs. Therefore, a major goal of this study was to establish the optimum parameters to condition cocaine CPA using trace conditioning. Interstimulus interval, drug dose, conditioning time and the type of conditioning apparatus have all been shown to impact the development of a CPP or avoidance [19, 25, 40, 41]. In the current study, we systematically varied cocaine dose, conditioning session length and conditioning chamber design (i.e., two‐chamber vs. three‐chamber) to determine the best set of parameters to induce cocaine CPA. We chose to keep the interstimulus interval short across all subjects, so that cocaine is administered immediately after removal from the conditioning apparatus, because this has been shown to produce the best CPA across drug types [25, 26]. Additionally, we investigated how the balance between dynorphin and enkephalin within the striatum predicts the development of cocaine CPA using trace conditioning. We used quantitative PCR to measure levels of the prodynorphin (*Pdyn*) and proenkephalin (*Penk*) gene and hypothesized that higher *Pdyn* in the ventral striatum would be correlated with cocaine CPA.

## 2 Materials and Methods

### 2.1 Animals

Male and female adult mice between 8 and 20 weeks of age were used for all experiments. To determine the best method to establish cocaine CPA, we used a variety of wild‐type mice, which consisted of in‐house bred Cre^−^ littermates or floxed mice from our transgenic mouse lines (all are on a C57BL/6J background). We used 103 mice: 41 *Adora2a‐Cre* ^*−/−*^ (RRID: MMRRC_034744-UCD; [42]), 2 MOR^f/f^ [43], 19 VGat:Cre (RRID: IMSR_JAX:028862) and 41 VGluT2:Cre (RRID: IMSR_JAX:016963). A subset of these (*Adora2a‐Cre* ^*−/−*^ = 24), along with six additional saline‐treated MOR^f/f^ mice to be used as controls, were used in a subsequent qPCR experiment to measure *Pdyn* and *Penk* in the striatum. To test whether a shifted balance between striatal enkephalin and dynorphin affects the development of cocaine CPA, we generated mice with a homozygous deletion of *Penk* from striatal dopamine D2 receptor–containing medium spiny neurons (D2‐*Penk*KO). This was achieved by crossing *Adora2a‐Cre* ^*+/−*^ and *Penk* ^*f/f*^ mice. We have used the *Adora2a‐Cre* line multiple times to selectively target these medium spiny neurons [44, 45], and this genetic cross produces a near complete reduction in enkephalin from striatal neurons [38, 46]. Furthermore, these D2‐*Penk*KO mice do not show alterations in striatal *Pdyn* expression [38]. For these experiments, 17 D2‐*Penk*KO and 17 *Adora2a‐Cre* ^*−/−*^ *Penk* ^*f/f*^ littermate controls were used. Mice were group‐housed in a temperature‐ and humidity‐controlled environment under 12:12 h light/dark cycle (lights on at 06:30 or 7:00) with food and water available ad libitum. Behavioural experiments were performed during the light cycle. All animal procedures were performed in accordance with guidelines from the Institutional Animal Care and Use Committees of the University of Texas Austin and Rutgers, the State University of New Jersey.

### 2.2 Drugs

Cocaine HCl was dissolved in sterile saline (0.9%) and administered intraperitoneally at a volume of 10 mL/kg. We tested a range of cocaine doses (15, 20 and 25 mg/kg) to determine the best dose to condition CPA. Previous studies report that rewarding doses of ethanol, nicotine and amphetamine were able to induce CPA using a trace conditioning procedure; thus, we selected cocaine doses known to induce place preference in previous experiments from our lab and others [23, 38, 41].

### 2.3 Conditioned Place Avoidance

#### 2.3.1 Apparatus

**FIGURE 1:**
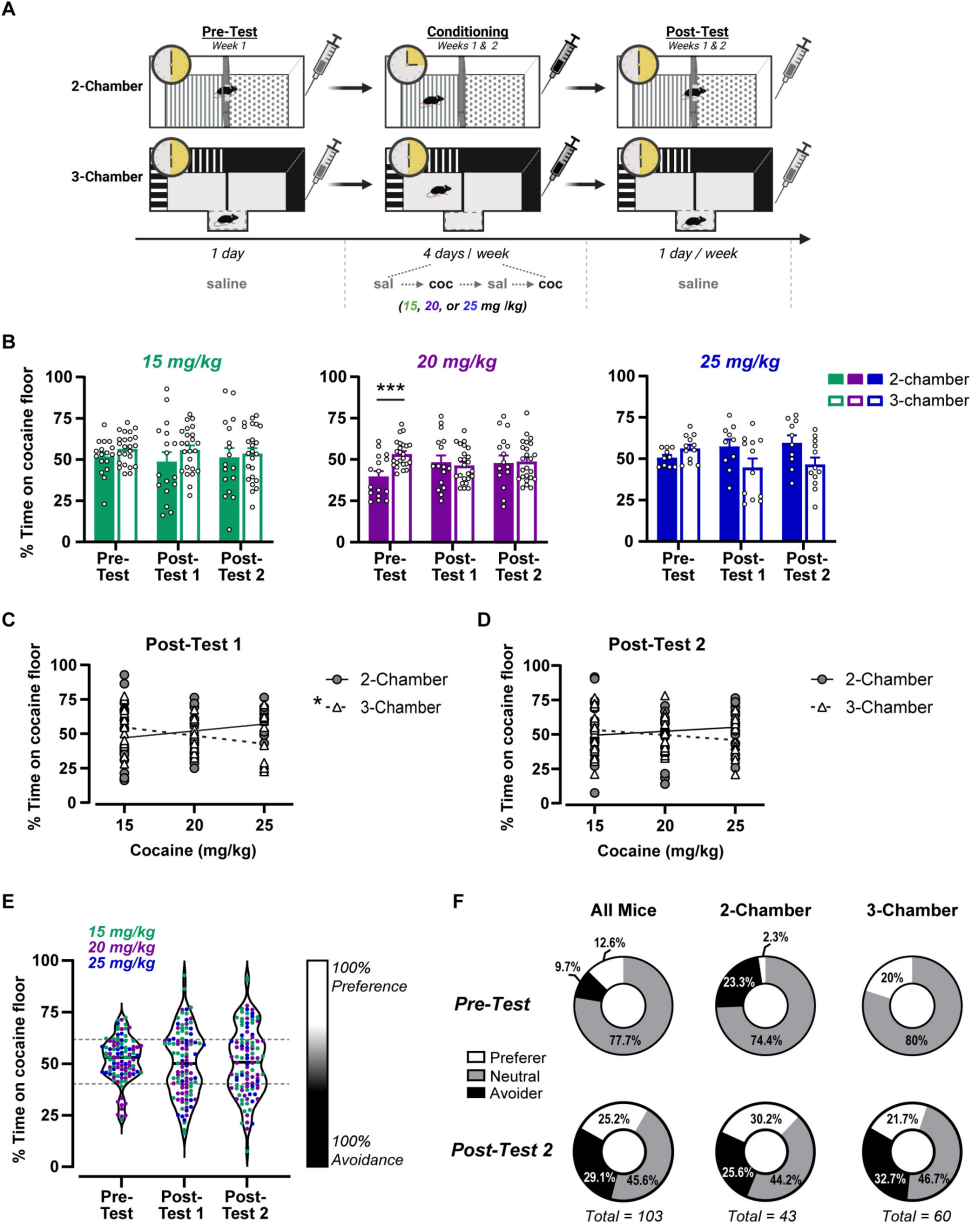
Cocaine dose, apparatus type and conditioning time do not influence the development of cocaine CPA. (A) Experimental timeline and schematic of conditioning apparatus types for the cocaine trace conditioning procedure. Mice were confined to one side of a two‐ or three‐chamber conditioning apparatus for 15 or 30 min, respectively. After removal, subjects received cocaine (15, 20 or 25 mg/kg; coc) or saline (sal) injection before returning to their home cage. Cocaine and saline conditioning were alternated over 2 weeks, with preference tests after conditioning Days 4 and 8. Created in BioRender. Dobbs, L. (2025) https://BioRender.com/yv7d6pc. (B) Mean percent time on the cocaine‐paired floor during pretest, Post‐Test 1 and Post‐Test 2 did not change across tests for any cocaine dose or apparatus type. Pretest values were lower for the two‐chamber compared with three‐chamber apparatus before conditioning (20 mg/kg; ***p < 0.001). (C, D) Linear regressions of cocaine dose versus preference score showed cocaine dose predicts the percent time on the cocaine floor only at Post‐Test 1 for the three‐chamber apparatus (*p < 0.05). (E) Violin plots show distribution of cocaine conditioning when collapsed across cocaine dose and apparatus type. Repeated measures ANOVA revealed no significant change in preference over testing, though increased variance was observed. Data were stratified based on one standard deviation of the pretest mean, which created a neutral zone (indicated by grey dashed lines; 40.28%–61.76%) flanked by subjects categorized as preferers (≥ 61.77%) and avoiders (≤ 40.27%). (F) The percentage of subjects in each strata at pretest are shown (top). Most mice were classified as neutral (grey) within the entire cohort (left) and in the two‐chamber (middle) and three‐chamber (right) apparatus. At Post‐Test 2 (bottom), there were approximately equal proportions of preferers (white) and avoiders (black), and this did not differ between apparatus type. Data are expressed as individual data points, mean ± SEM or a percent of total.

Mice were divided into two groups and received trace conditioning in either a two‐chamber (Med Associates) or three‐chamber (Stoelting) apparatus (Figure 1A). The two‐chamber apparatus consisted of two distinct contexts that differed by their tactile floor cue (grid and hole), which were separated by a guillotine door. The three‐chamber apparatus consisted of two contexts discriminated by tactile floor cues (smooth and textured) and visual stimuli (vertical white stripes and plain black walls), which were joined by a smaller connecting chamber.

#### 2.3.2 Procedure

##### 2.3.2.1 Experiment 1

To determine the optimum parameters to establish cocaine CPA using trace conditioning, we systematically varied cocaine dose, apparatus type and conditioning session length across two different protocols. Subjects were divided into two conditioning groups and received either 15‐min conditioning sessions in the two‐chamber apparatus or 30‐min conditioning sessions in the three‐chamber apparatus. For both groups, subjects were confined to one side of the conditioning chamber for their specific conditioning time, after which they received an injection of cocaine or saline and were returned to their home cage. Cocaine and saline conditioning sessions were alternated daily. An unbiased subject assignment procedure was used, and the drug‐context pairing, drug side and treatment order were counterbalanced. Subjects received a total of eight conditioning sessions (four cocaine and four saline) across 2 weeks. Preference tests were administered prior to conditioning (pretest) and after conditioning sessions 4 and 8 (Post‐Tests 1 and 2, respectively). Subjects were allowed to freely explore the entire apparatus during preference tests. All preference tests lasted 30 min, after which subjects received a saline injection and were returned to their home cage. Place preference and avoidance were determined by calculating the percent time spent on the cocaine‐paired floor. Different cocaine doses (15, 20 or 25 mg/kg) were tested in separate groups of subjects and for both conditioning groups.

##### 2.3.2.2 Experiment 2

To determine how the balance between striatal dynorphin and enkephalin affects the development of cocaine CPA, mice with a selective deletion of enkephalin from striatal D2‐MSNs (D2‐*Penk*KO) and littermate controls were trained in a trace conditioning procedure. Subjects were conditioned to 25‐mg/kg cocaine using a two‐chamber apparatus with a 15‐min conditioning session length as described in Experiment 1. All procedures and data were calculated as described above.

### 2.4 Quantitative Polymerase Chain Reaction

A subset of mice that had been conditioned with 25‐mg/kg cocaine (*Adora2a‐Cre* ^*−/−−*^ = 24) or received equal exposure to repeated saline injection (MOR^f/f^ = 6) were euthanized immediately after Post‐Test 2 to measure *Penk* and *Pdyn* using qPCR. The MOR^f/f^ saline controls, although not fully identical to nonfloxed wild‐type animals, are functionally wild‐type in the absence of Cre and ensure that potential effects of loxP insertion are equalized between Experiments 1 and 2. Mice were deeply anaesthetised with isoflurane, decapitated, the brain extracted and the dorsal and ventral striatum were dissected on ice. Tissue was homogenized and stored in RNA later until RNA was extracted (RNeasy, Qiagen), and cDNA was synthesized (iScript, BioRad). Relative mRNA expression of proenkephalin (*Penk*, Mm01212875_m1) and prodynorphin (*Pdyn*, Mm00457573_m1) was determined relative to the housekeeping gene β‐actin (*Actb*, Mm01205647_g1) using TaqMan Gene Expression Assays (ThermoFisher) utilizing a CFX384 Real‐Time System (initial hold at 95°C for 20 s, 40 cycles at 95°C for 1 s and 60°C for 20 s). Samples were run in triplicate, and negative controls were run in parallel. Relative expression of the genes of interest was calculated using the ΔΔCт method.

### 2.5 Statistical Analysis

Place conditioning data from the wild‐type dataset were quantified as the percent time spent on the cocaine‐paired floor and analysed using multifactorial ANOVAs with preference test as a repeated measure and chamber type and cocaine dose as between‐subject factors. To determine whether time spent in the neutral zone of the three‐chamber apparatus was driving place conditioning effects, separate two‐way ANOVAs were performed for each cocaine dose with zone as a between‐subject factor and test as a repeated measure. Sex effects were evaluated using two‐way ANOVA after collapsing place conditioning data across cocaine dose and chamber type. A simple linear regression was used to predict the effect of cocaine dose on cocaine avoidance. To better capture individual conditioning responses, we stratified subjects based on pretest variability using ±1 standard deviation from the pretest mean to define a ‘neutral’ zone at baseline (e.g., no preference). This method avoids arbitrary cutoffs (e.g., a median split) and ensures that classification as a preferer or avoider reflects a meaningful deviation from baseline and not preconditioning bias. Even so, a small number of subjects exhibited consistent pretest and post‐test conditioning (Experiment 1: 3 avoiders and 4 preferers out of 103 total; Experiment 2: 3 avoiders and 2 preferers out of 34 total); however, excluding these subjects did not alter the results (data not shown); thus, analyses were performed with all subjects included. Relative proportions of the preferer, avoider and neutral groups were analysed using Chi‐square analysis or Fisher's exact test when sample sizes were below five per group. The relationship between *Penk* and *Pdyn* mRNA expression with place conditioning was analysed using Pearson's correlation. Separate two‐way repeated measures ANOVA were performed for the ventral and dorsal striatum to determine how preference grouping affected relative opioid peptide gene expression. To analyse differences in cocaine avoidance in the D2‐*Penk*KO experiment and address the kurtosis observed in the dataset, a generalized linear model with an inverse Gaussian probability distribution was used to compare conditioning at pretest versus post‐test after 2 weeks of conditioning. Significant interactions from multifactorial datasets were followed up with two‐way ANOVA, simple main effects analyses and pairwise post hoc *t*‐tests when appropriate. Post hoc analyses were corrected for multiple comparisons using Sidak's. Data were analysed using Prism (GraphPad) or SPSS (IBM), and results were considered significant at an alpha of 0.05. All data are presented are individual values, percentages of total or mean ± SEM.

## 3 Results

### 3.1 Individual Differences in the Development of Cocaine CPA Are Not Predicted by Cocaine Dose or Apparatus Type

We first sought to determine the optimum parameters to establish cocaine CPA using a trace conditioning procedure. We varied the apparatus type (three‐chamber or two‐chamber), conditioning time (15 or 30 min) and cocaine dose (15, 20 or 25 mg/kg) between subjects (Figure 1A) and performed a three‐way RMANOVA to determine how these parameters affected the development of cocaine CPA. Overall, the mean percent time spent on the cocaine‐paired floor was fairly stable and did not significantly vary as a function cocaine dose or apparatus over testing (Test × Chamber × Dose: *F*_4,230_ = 2.14, *p* = 0.077; Figure 1B). Although there was an interaction between chamber and test (*F*_2,230_ = 7.1, *p* < 0.01), follow‐up post hoc analysis shows that this was driven by a difference at the pretest, rather than differential conditioning between the chamber types per se (two‐chamber vs. three‐chamber at pretest: *t*_357_ = 3.98, *p* < 0.001). Importantly, this pretest difference appears to be isolated to the group that later received 20‐mg/kg cocaine, suggesting that this preconditioning bias is a result of sampling error rather than the two‐chamber apparatus being generally biased (Figure 1B, middle). Cocaine dose also affected the percent time spent on the cocaine‐paired floor (Main effect of dose: *F*_2,115_ = 3.43, *p* < 0.05); however, post hoc analysis did not find significant differences between doses. To further probe this effect, we compared the linear regressions of cocaine dose versus preference score between the two chamber types at Post‐Tests 1 and 2. Cocaine dose predicted the percent time spent on the cocaine floor at Post‐Test 1 for the three‐chamber apparatus (*F*_1,58_ = 6.6, *p* < 0.05), but not the two‐chamber (*F*_1,40_ = 1.76, *p* = 0.19; Figure 1C). A higher cocaine dose was associated with greater avoidance of the cocaine‐paired floor in the three‐chamber apparatus; however, this relationship was small, and cocaine dose only accounted for 10% of the variation in preference (*R* ^2^ = 0.10). In contrast, cocaine dose was not predictive of the percent time spent on the cocaine‐paired floor at Post‐Test 2 for either apparatus type (Figure 1D). Thus, it appears that the highest cocaine dose (25 mg/kg) in the three‐chamber apparatus was slightly better at initially conditioning CPA. Importantly, place avoidance was not due to increased time spent in the nonconditioned ‘neutral’ zone (i.e., the small chamber connecting the two conditioning chambers), but rather less time spent in the cocaine zone and more time spent in the saline zone (Test × Zone: *F*_4,66_ = 5.7, *p* < 0.001; Table 1). Despite CPA being slightly stronger with this cocaine dose and the three‐chamber apparatus at Post‐Test 1, with more conditioning trials, neither cocaine dose nor apparatus type influenced the expression of CPA.

**TABLE 1:** Percent time spent per chamber at each preference test for 25‐mg/kg cocaine conditioning.

	Cocaine (25 mg/kg)	Saline	Unconditioned
Pretest	20.3 ± 0.6	16.1 ± 1.0	13.6 ± 0.7
Post‐Test 1	16.2 ± 1.9^a^	20.4 ± 2.1^b^	13.3 ± 1.0
Post‐Test 2	17.3 ± 1.5	20.1 ± 1.8^b^	12.6 ± 0.8

*Note:* Test × Chamber: *F*_4,66_ = 5.7, *p* < 0.001.

^a^ *p* = 0.06 versus pretest.

^b^ *p* = 0.09 versus pretest.

Given the minimal effect of cocaine dose and apparatus type on the development of cocaine CPA, we collapsed the data across these variables to increase our power to detect sex differences in cocaine CPA (Figure 1E). Cocaine preference did not differ between males and females at any preference test (*F*_1,95_ = 1.34, *p* = 0.25), and sex did not interact with cocaine dose (*F*_2,190_ = 1.00, *p* = 0.37); thus, all subsequent data analyses were performed collapsed across sex. When analysing the collapsed data, the mean percent time on the cocaine floor did not change across testing (*F*_2,204_ = 0.49, *p* = 0.61); however, the variability of the preference scores progressively increased between the pretest and post‐tests. This was reflected as a greater coefficient of variation at Post‐Test 2 (32.5%) compared with pretest (20.8%) and can be observed through the increasingly polarizing responses in Figure 1E. To examine these individual differences, preference scores at Post‐Test 2 were stratified into three categories by subtracting or adding one standard deviation (10.75, based on the pretest mean) from the Post‐Test 2 mean (51.02%). This created an upper threshold for Avoidance (≤ 40.27%), a lower threshold for Preference (≥ 61.77%) and a ‘neutral zone’ (40.28%–61.76%) (Figure 1F). We next compared the relative proportion of preferers, avoiders and neutral subjects, collapsed across apparatus type, and found a significant shift between pretest and Post‐Test 2 (*Χ* ^2^ [2, *n* = 103] = 51.08, *p* < 0.0001; Figure 1F). At Post‐Test 2, most subjects were classified as neutral (45.6%), and there were slightly more avoiders (29.1%) than preferers (25.2%). Between pretest and Post‐Test 2, the percentage of subjects expressing avoidance increased by threefold (from 9.7% to 29.1%). Although the percentage of preferers also increased, it only doubled between pretest and Post‐Test 2 (from 12.6% to 25.2%). Additionally, the relative proportions of preferers and avoiders were not different between apparatus types, suggesting that both apparatus types were equally good at conditioning cocaine CPA (*Χ* ^2^ [2, *n* = 103] = 1.08, *p* = 0.58). When taken together, these data indicate that cocaine causes bivalent responses that become increasingly prevalent at higher doses.

### 3.2 High Striatal Proenkephalin Relative to Prodynorphin Is Associated With Cocaine Avoidance

**FIGURE 2:**
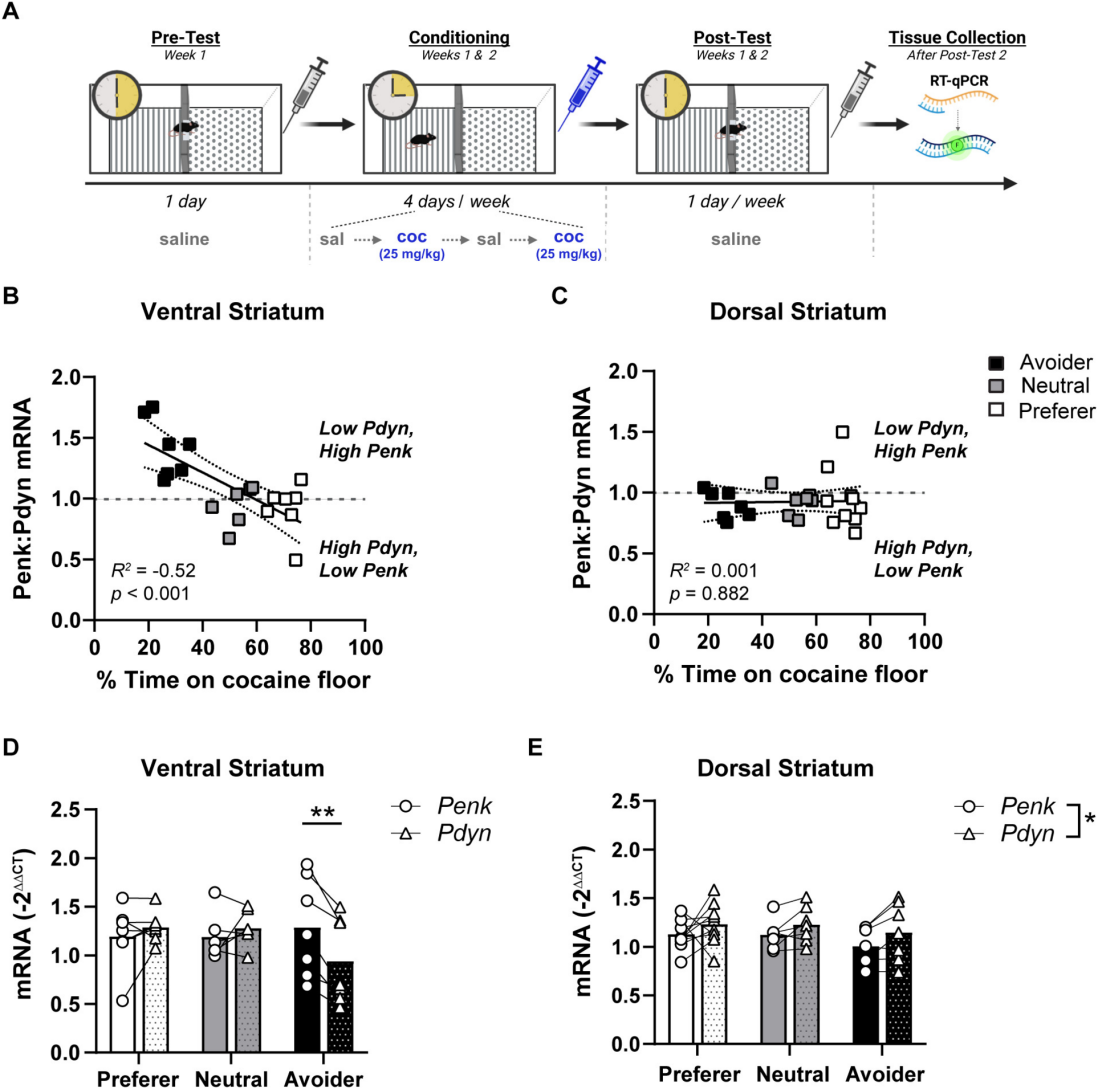
Cocaine CPA is associated with low prodynorphin levels in the ventral striatum. (A) Experimental timeline and apparatus schematic for cocaine trace conditioning. Mice were conditioned over 2 weeks in a two‐chamber apparatus for 15 min with 25‐mg/kg cocaine. Cocaine or saline was administered after removal from the conditioning chamber. Subjects were euthanized immediately following Post‐Test 2 and tissue was processed for RT‐qPCR. Created in BioRender. Dobbs, L. (2025) https://BioRender.com/cs6u8tv. (B, C) The ratio of *Penk*:*Pdyn* mRNA was negatively correlated with the percent time spent on the cocaine‐paired floor in the ventral (B), but not dorsal (C), striatum. Subjects expressing cocaine avoidance (black), neutral conditioning (grey) or preference (white) were stratified based on one standard deviation from the pretest mean (see Figure 1). (D) Subjects categorized as avoiders (black bars), but not preferers (white bars) or neutral subjects (grey bars), had lower *Pdyn* mRNA compared with *Penk* levels in the ventral striatum (Gene × Group interaction: *F*_2, 17_ = 9.7, *p* < 0.01; post hoc *t*‐test: **p < 0.01). (E) *Pdyn* was slightly higher than *Penk* levels for preferers, avoiders and neutral subjects in the dorsal striatum (Gene main effect, *p < 0.05). Data are expressed as individual data points or the mean.

We next investigated striatal opioid peptides as an underlying neurobiological mechanism responsible for biasing the development of cocaine preference or avoidance in a trace conditioning procedure. Within the striatum, MOR signalling is linked to motivation for food and cocaine [39, 47, 48]. In particular, augmenting enkephalin within the ventral striatum facilitates the acquisition of cocaine place preference [23]. Conversely, dynorphin‐mediated signalling through the KOR is aversive, though there is heterogeneity in this effect depending on the striatal subregion [35, 36]. We hypothesized that differential levels of striatal enkephalin relative to dynorphin may be a neurobiological correlate underlying the individual differences in the development of cocaine preference or avoidance following trace conditioning. To test this, we performed RT‐qPCR to measure *Penk* and *Pdyn* mRNA levels within the dorsal and ventral striatum of subjects following their completion of trace conditioning (25 mg/kg; two‐chamber apparatus; Figure 2A). We then compared the mRNA expression with each subject's percent time on the cocaine floor to determine if low *Penk* and high *Pdyn* were associated with cocaine CPA. Cocaine avoidance was not correlated with *Penk* mRNA levels in the dorsal or ventral striatum (Table 2). In contrast, *Pdyn* expression was positively associated with cocaine preference in the ventral, but not dorsal, striatum (*R* ^2^ = 0.2, *p* < 0.05; Table 2).

**TABLE 2:** Correlations (*R* ^2^) of percent time on the cocaine floor with striatal *Penk* and *Pdyn* mRNA.

%Time on cocaine floor	*Penk*, DS	0.095
	*Penk*, VS	0.0001
	*Pdyn*, DS	0.055
	*Pdyn*, VS	0.2004*

Abbreviations: DS, dorsal striatum; VS, ventral striatum.

* *p* < 0.05.

A correlation between cocaine preference and *Pydn*, but not *Penk*, suggests that a shift in the balance between these peptides may be influencing the development of cocaine preference versus avoidance. Indeed, dynorphin and enkephalin regulate the responsiveness of striatal medium spiny neurons and in this way play an important role in regulating striatal output, motor activity and motivated behaviour [30]. Thus, we correlated the *Penk*‐to‐*Pdyn* ratio with each subject's cocaine preference. Within the ventral striatum, a higher *Penk*:*Pdyn* ratio was negatively correlated with the percent time spent on the cocaine floor (*R* ^2^ = 0.52, *p* < 0.001; Figure 2B). In contrast, cocaine preference was not correlated with the *Penk*:*Pdyn* ratio in the dorsal striatum (Figure 2C).

The strong negative association between ventral striatum *Penk*:*Pdyn* with cocaine preference indicates that the balance of these peptides may be important for regulating cocaine avoidance. However, this analysis cannot ascertain whether this is due to higher *Penk*, lower *Pdyn* or a combination of the two. To address this, we stratified each subject's mRNA expression into preferers, avoiders and neutral groups using one standard deviation of the pretest mean (Figure 1E). Within the ventral striatum, the relationship between *Penk* and *Pdyn* mRNA expression depended on the preference group (Gene × Group: *F*_2,17_ = 9.7, *p* < 0.01; Figure 2D). Subjects categorized as avoiders had higher *Penk* levels relative to *Pdyn* (*t*_17_ = 4.32, *p* < 0.01). In contrast, there was no difference between *Penk* and *Pdyn* for subjects that preferred cocaine or were neutral. Additionally, although mean levels of *Pdyn* were lower for avoiders compared with preferers or neutral subjects, this comparison did not reach statistical significance. Within the dorsal striatum, the balance between *Penk* and *Pdyn* was similar across avoiders, preferers and neutral subjects, with *Pdyn* being slightly elevated relative to *Penk* across all groups (Gene: *F*_1,21_ = 7.03, *p* < 0.05; Figure 2E). This is consistent with the literature indicating that cocaine increases striatal *Pdyn* mRNA and peptide levels [29, 49, 50, 51, 52, 53, 54], particularly in the dorsal striatum [55]. In addition, levels of the control gene, beta actin, were consistent between treatment groups, indicating that differences in *Pdyn* or *Penk* expression were not due to non‐specific changes in gene expression (saline vs. cocaine: 20.09 ± 0.14, 19.95 ± 0.09; unpaired *t*‐test: *t*_113_ = 0.78, *p* = 0.44). Taken together, cocaine CPA is associated with lower *Pdyn* levels relative to *Penk* selectively in the ventral striatum.

### 3.3 Higher Relative *Pdyn* Within the Striatum Prevents Development of Cocaine Avoidance

Although the qPCR results are informative, they cannot preclude whether differences in *Pdyn* and *Penk* existed prior to conditioning. Thus, we next tested whether the lower *Pdyn* relative to *Penk* levels in the ventral striatum of avoiders represented a causal, pre‐existing neural signature that biases towards the development of CPA in a trace conditioning procedure. To address this, we took advantage of a cell‐selective knockout mouse line in our laboratory that lacks *Penk* in striatal D2‐MSNs (D2‐*Penk*KO; [38]). These mice show a near complete lack of *Penk* in the striatum [38] and therefore have a pre‐existing shift in the striatal *Penk*:*Pdyn* ratio that favours a higher *Pdyn* tone relative to *Penk*. Given our observations that (1) higher *Penk* relative to *Pdyn* is associated with cocaine avoidance and (2) higher *Pdyn* relative to *Penk* is associated with cocaine preference, we hypothesized that D2‐*Penk*KO mice would be more resistant to developing cocaine CPA. Additionally, since wild‐type mice show a wide distribution of preferers versus avoiders, we speculated that a difference between the D2‐*Penk*KOs and littermate controls would likely be best reflected as a shift in the relative proportions of preferers, avoiders and neutral mice rather than in differences in the mean percent time on the cocaine floor per se.

**FIGURE 3:**
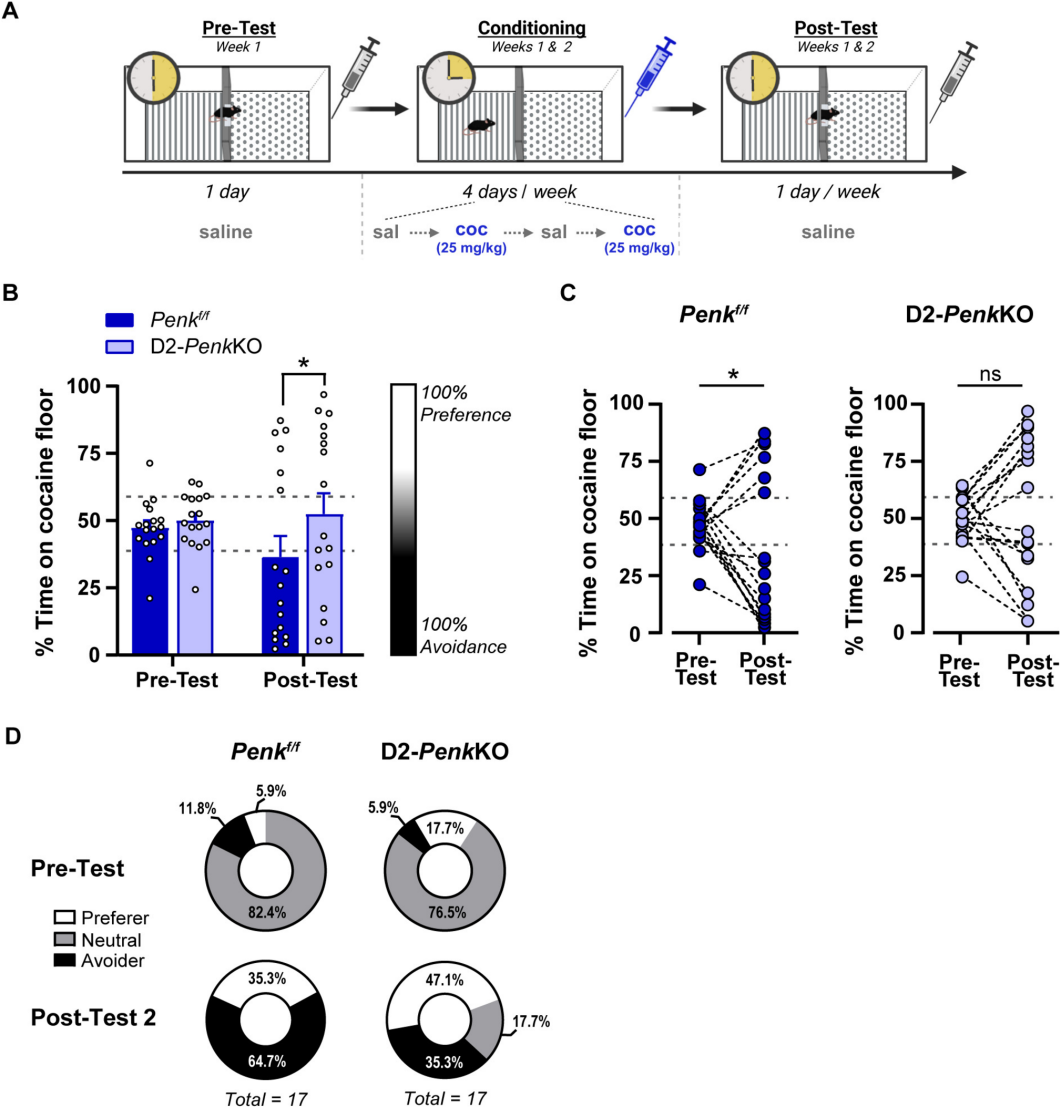
High relative striatal prodynorphin prevents development of cocaine CPA. (A) Experimental timeline and apparatus schematic for cocaine trace conditioning. Mice lacking proenkephalin from D2 receptor containing medium spiny neurons (D2‐*Penk*KO) and littermate controls (*Penk* ^*f/f*^) were conditioned over 2 weeks in a two‐chamber apparatus for 15 min with 25‐mg/kg cocaine. Cocaine or saline was administered after removal from the conditioning chamber. Created in BioRender. Dobbs, L. (2025) https://BioRender.com/crjxcp3. (B) *Penk* ^*f/f*^ subjects had lower percent time spent on the cocaine‐paired floor compared with D2‐*Penk*KOs at the post‐test (*p < 0.05). Data were stratified based on one standard deviation of the pretest mean, which created a neutral zone (indicated by grey dashed lines; 38.6%–59%) flanked by subjects categorized as preferers (≥ 59%) and avoiders (≤ 38.5%). (C) Post hoc follow up analysis of Post‐Test 2 compared with pretest indicated *Penk* ^*f/f*^ developed a cocaine CPA (*p < 0.05), whereas D2‐*Penk*KO showed neither preference nor aversion (ns = not significant). (D) The percentage of subjects in each strata at pretest are shown (top), and most subjects were categorized as neutral for both genotypes. At Post‐Test 2 (bottom), slightly more *Penk* ^*f/f*^ subjects were categorized as avoiders (black) than D2‐*Penk*KO, which had slightly more preferers (white), but this was not significant. Data are expressed as individual data points, mean ± SEM or a percent of total.

The distribution of preference scores in the D2‐*Penk*KO and littermate controls was not normally distributed and exhibited significant kurtosis for pretest and Post‐Test 2. Therefore, we used a generalized linear model (Wald's Chi‐square) with an inverse Gaussian distribution to approximate the distribution and analyse the change in preference between pretest and Post‐Test 2. We observed that the percent time on the cocaine floor changed across testing depending on the genotype (Genotype × Test: Wald Chi‐square: 5.73, df = 1, *p* < 0.05; Figure 3B,C), despite no difference between genotype in the pretest (Figure 3C). Post hoc follow‐up analysis confirmed that by Post‐Test 2, *Penk* ^*f/f*^ controls had developed a significant cocaine avoidance (*p* < 0.05) compared with the pretest. In contrast, cocaine CPA was mitigated in D2‐*Penk*KOs (*p* = 0.12) (Figure 3C). Moreover, the difference between genotypes was also significant at Post‐Test 2 (*p* < 0.05), but not at pretest, suggesting that a higher *Pdyn* tone relative to *Penk* prevented the development of cocaine avoidance. We next evaluated the proportion of avoiders and preferers within each genotype by stratifying subjects based on pretest variability using ± 1 standard deviation from the pretest mean ($\bar{X}$ = 48.7, *σ* = 10.2) to define a ‘neutral’ zone at baseline (e.g., no preference; 38.6%–59%). Subjects with preference above or below this strata were categorized as preferers (≥ 59%) or avoiders (≤ 38.5%), respectively. The mean conditioning score for *Penk* ^*f/f*^ controls (36.5%), but not for D2‐*Penk*KOs (52.4%), fell inside the region of avoidance. At pretest, the relative proportions of preferers, avoiders and neutral were not different between genotypes, and most subjects fell into the neutral range (Fisher's exact test; *p* = 0.69; Figure 3D, top). At Post‐Test 2, both genotypes showed a significant shift in the relative proportions of these groups (Fisher's exact test; *Penk* ^*f/f*^: *p* < 0.0001; D2‐*Penk*KO: *p* < 0.01). Although the proportions were not different between genotypes (Fisher's exact test; *p* = 0.12; Figure 3D, bottom), the low per strata sample sizes may limit the ability to detect differences. Taken together, these data suggest that the balance between striatal *Penk* and *Pdyn* may influence the development of cocaine preference or avoidance using a trace conditioning procedure.

## 4 Discussion

### 4.1 Optimum Parameters for Cocaine CPA

In this investigation, we sought to establish the optimum parameters to condition cocaine place avoidance using a trace conditioning procedure and to investigate the striatal opioid milieu as a neurobiological correlate of cocaine CPA. Previous research has shown CPA develops following trace conditioning to ethanol, amphetamines and nicotine. However, despite self‐report and preclinical data indicating that cocaine has aversive qualities associated with intoxication, CPA to cocaine using trace conditioning has not been established. Moreover, CPA for cocaine has traditionally proven more difficult in mouse models, as compared with work in rats [6]. We tested different cocaine doses, conditioning session times and apparatus types, which are variables previously shown to influence the development of drug conditioning. We observed a large variability in cocaine conditioning, with roughly equal proportions of subjects developing preference and avoidance. Quite to our surprise, the propensity to condition avoidance was not reliably predicted by any of the parameters we manipulated, nor was backwards conditioning more effective (unpublished observations). Additionally, sex was not a factor in determining the likelihood of conditioning cocaine place avoidance. Although conditioning time varied between the two‐chamber and three‐chamber apparatuses, there was no difference between these experimental results, which speaks to the robust nature and generalizability of these findings.

The small effect of cocaine dose on cocaine CPA was specific to the three‐chamber apparatus and did not persist past 1 week of conditioning. It is possible a higher dose (i.e., > 25 mg/kg) may be more acutely aversive and therefore condition a larger, more enduring CPA. Using a simultaneous conditioning procedure, most doses of methamphetamine resulted in CPA, whereas only very low doses produced conditioned preferences [56]. To our knowledge, though, there are no reports showing an inverted‐U dose–response for cocaine preference with simultaneous conditioning. Thus, it is unclear whether the balance between reward and aversion shifts towards more aversion with increasing cocaine dose, or perhaps whether the aversive qualities of cocaine require a longer history of drug use. It should be noted, however, that acutely aversive drug doses do not appear to be necessary because previous experiments use rewarding drug doses to induce CPA in trace conditioning [25, 26, 27].

Mean CPA was slightly larger when subjects were conditioned using a 30‐min session time within the three‐chamber apparatus. In simultaneous conditioning procedures, conditioning session time does not affect cocaine place preference [41]. It is conceivable that longer conditioning times may lead to a stronger memory of the CS when the drug is administered and thus a better CPA; however, the small effect in the current dataset suggests that this is not the case. Consistent with this, ethanol CPA develops with only a 5‐min conditioning length [25]. One advantage of the three‐chamber apparatus is the distinct presentation of conditioned cues. However, data from three‐chamber conditioning apparatuses, although commonly used, can also be difficult to interpret if the time spent in the unconditioned ‘neutral’ chamber changes significantly after conditioning. Subjects may spend more time in the unconditioned chamber because of its relative novelty, and drugs that block the habituation to novelty may enhance this effect [57]. In the current experiment, time spent in unconditioned chamber did not significantly change after conditioning, indicating that the preference and avoidance observed were not the result of shifts in novelty perception of the unconditioned chamber. Taken together, the trace conditioning procedure yields fairly equal cocaine conditioning between the two‐chamber and three‐chamber apparatuses, indicating neither is preferential for generating cocaine CPA.

### 4.2 Interpretations of Cocaine CPA in Trace Conditioning

Several potential interpretations have been posited to explain the development of drug CPA using trace conditioning, including interfering with habituation of neophobia [26], the drug‐paired context becoming a conditioned inhibitor potentially by conditioning a ‘drug wanting’ state [58] or by conditioning an initial, transient aversive drug effect [25]. If the drug interferes with habituation of neophobia associated with the conditioning chamber, CPA may represent contextual neophobia rather than an aversive aspect of the drug per se. However, this interpretation does not explain why subjects express place preference when the same drug is administered in a simultaneous conditioning procedure. Thus, although we cannot rule this out for other drugs with dissociative properties, this appears to be an unsatisfactory explanation for cocaine CPA in the current study.

In the case of the drug‐paired context becoming a Pavlovian conditioned inhibitor, the conditioning chamber is thought to elicit avoidance because it predicts the absence of the rewarding drug sensation and a ‘drug wanting’ state [59, 60]. However, morphine and diazepam fail to condition a place avoidance (or preference) in a trace conditioning procedure [61, 62, 63, 64]. This does not appear due to subthreshold dosing because the same doses elicited place preference using a simultaneous conditioning procedure [61, 64]. Additionally, if the delay of drug reward was indeed driving the CPA, one would expect the CPA to maintain, and perhaps intensify, with increasing delay between the CS and US. When increasing delays were introduced in ethanol trace conditioning experiments, though, CPA was abolished [25]. In contrast, studies using a conditioned taste aversion procedure report robust aversion with a 30‐min delay between the CS and cocaine self‐administration [60]. However, despite similar CS–US temporal dynamics between trace conditioning and conditioned taste aversion experiments, the conditioned responses to contextual versus gustatory stimuli can be different. For instance, morphine administration following consumption of a novel tastant within a conditioning chamber results in taste, but not place, avoidance [61]. Notably, stronger relationships between the qualities of specific cues and the subsequent reinforcers have long been shown to support stronger conditioning [65]. Thus, it is possible that the ability of a stimulus to support Pavlovian associations with motivational drug effects depends on the stimulus modalities and specific physiologic drug response. However, coupling these conditioning discrepancies with evidence that CPA degrades with increasing delays makes the conditioned inhibitor explanation of CPA less likely. Moreover, conditioned inhibition does not reasonably explain why in the current study approximately equal proportions of subjects developed preference and avoidance.

The development of CPA in trace conditioning may also result from subjects experiencing a transient aversive state following cocaine injection, which becomes paired with the preceding context. The onset of a longer‐lasting rewarding affective state is thought to occur afterwards and is more likely paired with the home cage environment. This transient aversion may stem from various sources such as the transition from sobriety to intoxication, peritoneal irritation or an aversive state associated with cocaine intoxication. We suspect aversion due to state transition or peritoneal irritation is unlikely. Only a subset of subjects developed CPA in the current study and not all intoxicating drugs condition avoidance. Further, there is not convincing evidence for peritoneal irritation at the cocaine doses used in the current study and, if present, this would occur equally for the saline‐paired chamber. Evidence from human cocaine users [8, 9, 10] and rodent models [11, 12, 13] indicate that cocaine elicits an anxiogenic state immediately after administration in a subset of subjects, supporting the hypothesis that CPA in the current study resulted from an acutely aversive state associated with cocaine intoxication. How long this aversive state lasts is not known, but it is likely a combination of pharmacokinetic and pharmacodynamic effects. Further, although only a subset of subjects conditioned avoidance, it recapitulates, to an astonishing degree, the rate of human cocaine users who report experiencing a negative affective state associated with cocaine use (29.1% in our mouse population vs. 24% in humans) [10]. Thus, the culmination of evidence suggests that cocaine CPA likely results from a Pavlovian association between a transient, cocaine‐induced negative affective state and the preceding context.

### 4.3 Striatal Opioids as a Mechanism Influencing the Development of Cocaine Avoidance

Dynorphin‐mediated KOR signalling within the ventral striatum is associated with cocaine withdrawal [66] and pain‐induced negative affect [67], and withdrawal from chronic cocaine increases dynorphin levels within the striatum [30]. This heightened dynorphin tone is thought to act as a negative reinforcer, because KOR activation incites stress‐induced reinstatement of cocaine seeking [33, 65] and blockade of KORs attenuates it [32, 68]. Based on this literature, we hypothesized that cocaine CPA would be associated with high striatal dynorphin. Conversely, subjects expressing cocaine CPA had lower levels of striatal *Pdyn* compared with preferer and neutral subjects, and this effect was isolated to the ventral striatum. Together, these data suggest that striatal dynorphin signalling may have opposing roles in mediating aversion associated with acute intoxication versus cocaine withdrawal. Considering that KOR‐mediated signalling facilitates cocaine relapse after withdrawal [69], the association between low striatal *Pdyn* levels and cocaine CPA in the current data support the interpretation that avoidance is due to a transient cocaine aversion, rather than a ‘drug wanting’ state.

The finding that mice with higher relative striatal dynorphin to enkephalin (i.e., D2‐*Penk*KO) have greater cocaine seeking is consistent with the literature and supports the hypothesis that high striatal dynorphin tone drives cocaine seeking. Thus, place preference may reflect the experience of an aversive ‘drug wanting’ state mediated by high striatal dynorphin. This is supported by the current data in D2‐*Penk*KO mice since, even though *Pdyn* levels were not directly measured in the current study, our prior findings indicate they have higher *Pdyn*:*Penk* mRNA levels compared with littermate controls [38]. Consistent with this, the non‐selective opioid antagonist naloxone blocks expression of cocaine preference in female D2‐*Penk*KO mice and littermate controls [38]. Whether this effect was mediated by dynorphin or another opioid peptide, though, is unclear. This is an important consideration because enkephalin tone and MOR‐mediated signalling within the ventral striatum also facilitate the acquisition and expression of cocaine place preference [23]. Another potential interpretation is that D2‐*Penk*KO mice have a generally potentiated cocaine reward, though our previous work indicates that D2‐*Penk*KO mice acquire cocaine place preference normally in a simultaneous conditioning procedure [38]. Dynorphin‐mediated ‘drug wanting’ may be a less probable explanation for cocaine preference in the wild‐type mice from the current study because *Pdyn* levels for preferers were similar to that of neutral and avoiders. Thus, KOR‐mediated signalling was likely not driving cocaine seeking in these subjects. It is possible that place preference is a result of unmasking cocaine reward due to a blunted transient aversion. This is also unlikely, though, since rewarding doses of opiates and benzodiazepines do not condition avoidance or preference in a trace conditioning procedure [61, 63, 64] but do result in preference in simultaneous conditioning [61]. Taken together, these data suggest that although enkephalin and dynorphin both drive cocaine seeking, they do so through opposing motivational mechanisms. Moreover, our data suggest that pre‐existing heightened striatal dynorphin relative to enkephalin, as in the D2‐*Penk*KO mice, is a causal mechanism rendering subjects more sensitive to the transient aversion associated with cocaine intoxication.

### 4.4 Limitations to the Current Study

The selectivity of the association between dynorphin and cocaine CPA for the ventral striatum is consistent with previous reports indicating that dynorphin signalling specifically within the ventral striatum is associated with cocaine withdrawal. However, work by Al‐Hasani and colleagues [35] indicates even further subregion specificity related to dynorphin‐mediated CPA, with KOR signalling within the ventral, but not dorsal, nucleus accumbens shell conditioning place avoidance. Although it is unclear if this subregion distinction is conserved regarding dynorphin's role in cocaine conditioning, the brain dissections and qPCR used in the current study cannot distinguish between these subregions and thus is a limitation of this approach. Additionally, the use of *Penk* and *Pdyn* mRNA expression analysis to probe differences in the endogenous opioid milieu, although highly quantitative, may not completely correlate with enkephalin and dynorphin peptide levels. The use of semi‐quantitative approaches to measure peptide levels, such as immunofluorescence, or real‐time detection of peptide release using sensors may complement this approach in future studies. Lastly, it remains unclear whether the qPCR results represent pre‐existing differences that lead to CPA, or whether the differences in *Penk* and *Pdyn* mRNA expression developed alongside cocaine conditioning. Although we unfortunately cannot sample the brain prior to conditioning, the culmination of our results suggests that both interpretations may be possible. The gradual development of CPA and preference may indicate that a shift in dynorphin tone occurs because of cocaine exposure. This is consistent with time course studies showing striatal *Pdyn* levels increase in order to restrain cocaine‐induced activation of D1‐MSNs [30]. However, our finding that D2‐*Penk*KOs are resistant to developing cocaine CPA may also indicate that a pre‐existing shift in the balance between striatal dynorphin and enkephalin can predispose development of cocaine CPA. Future experiments capable of directly assessing striatal dynorphin tone in real time or using calcium imaging to track the activity of D1‐ and D2‐MSNs will undoubtedly be beneficial in addressing questions such as these.

Our findings suggest that enkephalin and dynorphin within the striatum are important for the expression of conditioned rewarding and aversive aspects of cocaine. It is tempting to speculate that levels of dynorphin itself might be most essential to the expression of place preference or avoidance because avoiders showed lower *Pdyn* levels compared with neutral and preferer mice, with no differences in *Penk* levels. However, because shifts in *Pdyn* fundamentally alter the balance with *Penk*, these data cannot distinguish whether absolute peptide level or balance between the peptides is responsible for the effect. Similarly, genetically deleting *Penk* from striatal MSNs also inherently shifts the balance to favour dynorphin signalling within the striatum. Future experiments using intracranial administration of short‐acting KOR antagonists during preference testing will help disentangle how acute changes in dynorphin‐mediated signalling affect expression of CPA without inducing long‐term alterations in the enkephalin‐dynorphin balance.

When testing different parameters to establish cocaine CPA in Experiment 1, we observed a slightly larger CPA with the 30‐min conditioning session. However, since this conditioning time was only tested in the three‐chamber apparatus, we cannot differentiate whether the timing or apparatus type was the main driver for this effect. For Experiment 2, we used the highest cocaine dose to establish cocaine CPA. Interestingly, a larger proportion of control mice developed CPA in Experiment 2 than in the initial experiment, which may in part have driven the genotype difference. Considering the smaller sample size in Experiment 2, we cannot rule out the possibility that sampling error contributed to the more robust CPA in controls. Although it is tempting to compare the D2‐*Penk*KO mice to control mice from Experiment 1, the most appropriate comparison is the littermate controls run in parallel. Additionally, it is not clear whether this genotype difference would persist across other cocaine doses.

## 5 Conclusions

Cocaine trace conditioning is a good approach to observe the individual differences in the development of cocaine preference and avoidance and a means to identify subjects that are more vulnerable to the brief aversive state that occurs following the administration of a rewarding cocaine dose. This transient aversive state can curb future cocaine use, suggesting an increased sensitivity to the aversive component may be protective against developing cocaine abuse. Conversely, these individuals may be more prone to coabuse opioids to counteract this aversive state. The balance between striatal enkephalin and dynorphin may represent a neurobiological mechanism underlying the vulnerability to the transient aversion induced by cocaine. Future experiments can leverage this procedure and these kinds of data to further probe the causal role of striatal opioids in this behaviour and identify new mechanisms that are potentially protective against cocaine abuse or even predictive of cocaine‐opiate coabuse.

## Author Contributions

Conceptualization and methodology: D.J.B. and L.K.D. Investigation and formal analysis: D.J.B., L.K.D., A.N. and P.K. Writing – original draft: L.K.D. and A.N. Writing – review and editing: D.J.B., L.K.D. and A.N. Supervision, funding acquisition and resources: D.J.B. and L.K.D.

## Ethics Statement

All animal procedures were performed in accordance with the guidelines from the Institutional Animal Care and Use Committees of the University of Texas Austin and Rutgers, the State University of New Jersey.

## Conflicts of Interest

The authors declare no conflicts of interest.

## Supporting information


**Figure S1** Development of cocaine preference or avoidance is not determined by a preconditioning bias. (A–C) Data were stratified based on one standard deviation of the pretest mean, which created a neutral zone (indicated by dashed, grey lines; 40.28%–61.76%), flanked by subjects categorized as preferers (≥ 61.77%) and avoiders (≤ 40.27%). Conditioning scores at pretest and Post‐Test 2 are shown paired for each subject conditioned with 15 mg/kg (A), 20 mg/kg (B) or 25 mg/kg (C) cocaine. Data are expressed as individual values. Related to Figure 1.

## Data Availability

The data that support the findings of this study are available from the corresponding author upon reasonable request.

## References

[1] M. Orndorff , G. M. Shipp , J. M. Kerver , S. J. Ondersma , and O. Alshaarawy , “Trends in Cocaine Use Among United States Females of Reproductive Age, 2005–2019,” American Journal on Addictions 33 (2024): 313–319, 10.1111/ajad.13502. 37924245

[2] L. Brandt , T. Chao , S. D. Comer , and F. R. Levin , “Pharmacotherapeutic Strategies for Treating Cocaine Use Disorder—What Do We Have to Offer?,” Addiction 116 (2021): 694–710, 10.1111/add.15242. 32888245 PMC7930140

[3] D. J. Barker , S. J. Simmons , L. C. Servilio , et al., “Ultrasonic Vocalizations: Evidence for an Affective Opponent Process During Cocaine Self‐Administration,” Psychopharmacology 231 (2014): 909–918, 10.1007/s00213-013-3309-0. 24197178 PMC3989366

[4] A. Ettenberg , M. A. Raven , D. A. Danluck , and B. D. Necessary , “Evidence for Opponent‐Process Actions of Intravenous Cocaine,” Pharmacology Biochemistry and Behavior 64 (1999): 507–512, 10.1016/S0091-3057(99)00109-4. 10548263

[5] F. H. Gawin and H. D. Kleber , “Abstinence Symptomatology and Psychiatric Diagnosis in Cocaine Abusers. Clinical Observations,” Archives of General Psychiatry 43 (1986): 107–113, 10.1001/archpsyc.1986.01800020013003. 3947206

[6] T. C. Jhou , C. H. Good , C. S. Rowley , et al., “Behavioral/Cognitive Cocaine Drives Aversive Conditioning via Delayed Activation of Dopamine‐Responsive Habenular and Midbrain Pathways,” Journal of Neuroscience 33, no. 17 (2013): 7501–7512, 10.1523/JNEUROSCI.3634-12.2013. 23616555 PMC3865501

[7] R. Sinha , T. Fuse , L. R. Aubin , and S. S. O'Malley , “Psychological Stress, Drug‐Related Cues and Cocaine Craving,” Psychopharmacology 152 (2000): 140–148, 10.1007/s002130000499. 11057517

[8] J. C. Anthony , A. Y. Tien , and K. R. Petronis , “Epidemiologic Evidence on Cocaine Use and Panic Attacks,” American Journal of Epidemiology 129 (1989): 543–549, 10.1093/oxfordjournals.aje.a115166. 2916547

[9] R. Kalayasiri , A. Sughondhabirom , R. Gueorguieva , et al., “Self‐Reported Paranoia During Laboratory “Binge” Cocaine Self‐Administration in Humans,” Pharmacology, Biochemistry, and Behavior 83 (2006): 249–256, 10.1016/j.pbb.2006.02.005. 16549106

[10] S. Williamson , M. Gossop , B. Powis , P. Griffiths , J. Fountain , and J. Strang , “Adverse Effects of Stimulant Drugs in a Community Sample of Drug Users,” Drug and Alcohol Dependence 44 (1997): 87–94, 10.1016/S0376-8716(96)01324-5. 9088780

[11] R. Rogerio and R. N. Takahashi , “Anxiogenic Properties of Cocaine in the Rat Evaluated With the Elevated Plus‐Maze,” Pharmacology, Biochemistry, and Behavior 43 (1992): 631–633, 10.1016/0091-3057(92)90203-r. 1438502

[12] P. Simon , R. Dupuis , and J. Costentin , “Thigmotaxis as an Index of Anxiety in Mice. Influence of Dopaminergic Transmissions,” Behavioural Brain Research 61 (1994): 59–64, 10.1016/0166-4328(94)90008-6. 7913324

[13] X. M. Yang , A. L. Gorman , A. J. Dunn , and N. E. Goeders , “Anxiogenic Effects of Acute and Chronic Cocaine Administration: Neurochemical and Behavioral Studies,” Pharmacology, Biochemistry, and Behavior 41 (1992): 643–650, 10.1016/0091-3057(92)90386-t. 1584846

[14] A. Ettenberg and T. D. Geist , “Animal Model for Investigating the Anxiogenic Effects of Self‐Administered Cocaine,” Psychopharmacology 103 (1991): 455–461, 10.1007/BF02244244. 2062985

[15] A. Ettenberg , “Opponent Process Properties of Self‐Administered Cocaine,” Neuroscience & Biobehavioral Reviews 27 (2004): 721–728, 10.1016/j.neubiorev.2003.11.009. 15019422

[16] T. D. Geist and A. Ettenberg , “Concurrent Positive and Negative Goalbox Events Produce Runway Behaviors Comparable to Those of Cocaine‐Reinforced Rats,” Pharmacology Biochemistry and Behavior 57 (1997): 145–150, 10.1016/S0091-3057(96)00300-0. 9164565

[17] H. Li , M. Eid , D. Pullmann , Y. S. Chao , A. A. Thomas , and T. C. Jhou , “Entopeduncular Nucleus Projections to the Lateral Habenula Contribute to Cocaine Avoidance,” Journal of Neuroscience 41, no. 2 (2021): 298–306, 10.1523/JNEUROSCI.0708-20.2020 33214316. 33214316 PMC7810656

[18] J. Parrilla‐Carrero , M. Eid , H. Li , Y. S. Chao , and T. C. Jhou , “Synaptic Adaptations at the Rostromedial Tegmental Nucleus Underlie Individual Differences in Cocaine Avoidance Behavior,” Journal of Neuroscience 41 (2021): 4620–4630, 10.1523/JNEUROSCI.1847-20.2021. 33753546 PMC8260244

[19] T. M. Tzschentke , “Measuring Reward With the Conditioned Place Preference Paradigm: A Comprehensive Review of Drug Effects, Recent Progress and New Issues,” Progress in Neurobiology 56 (1998): 613–672. 9871940 10.1016/s0301-0082(98)00060-4

[20] T. M. Tzschentke , “Measuring Reward With the Conditioned Place Preference (CPP) Paradigm: Update of the Last Decade,” Addiction Biology 12 (2007): 227–462, 10.1111/j.1369-1600.2007.00070.x. 17678505

[21] M. T. Bardo , J. K. Rowlett , and M. J. Harris , “Conditioned Place Preference Using Opiate and Stimulant Drugs: A Meta‐Analysis,” Neuroscience & Biobehavioral Reviews 19 (1995): 39–51, 10.1016/0149-7634(94)00021-R. 7770196

[22] C. L. Cunningham , D. R. Niehus , D. H. Malott , and L. K. Prather , “Genetic Differences in the Rewarding and Activating Effects of Morphine and Ethanol,” Psychopharmacology 107 (1992): 385–393. 1352057 10.1007/BF02245166

[23] K. Z. Dai , I. B. Choi , R. Levitt , et al., “Dopamine D2 Receptors Bidirectionally Regulate Striatal Enkephalin Expression: Implications for Cocaine Reward,” Cell Reports 40 (2022): 111440, 10.1016/j.celrep.2022.111440. 36170833 PMC9620395

[24] L. K. Dobbs and C. L. Cunningham , “The Role of the Laterodorsal Tegmental Nucleus in Methamphetamine Conditioned Place Preference and Locomotor Activity,” Behavioural Brain Research 265 (2014): 198–202, 10.1016/j.bbr.2014.02.021. 24569009 PMC4020626

[25] C. L. Cunningham and D. M. Okorn , “Interstimulus Interval Determines Whether Ethanol Produces Conditioned Place Preference or Aversion in Mice,” Animal Learning & Behavior 25, no. 1 (1997): 31–42, 10.3758/BF03199022.

[26] P. J. Fudala and E. T. Iwamoto , “Conditioned Aversion After Delay Place Conditioning With Nicotine,” Psychopharmacology 92 (1987): 376–381. 3114791 10.1007/BF00210847

[27] P. J. Fudala and E. T. Iwamoto , “Conditioned Aversion After Delay Place Conditioning With Amphetamine,” Pharmacology, Biochemistry, and Behavior 35 (1990): 89–92. 2315374 10.1016/0091-3057(90)90209-z

[28] J. E. Purdy , M. R. Markham , B. L. Schwartz , and W. C. Gordon , Learning and Memory, 2nd ed. (Wadsworth/Thomson Learning, 2001).

[29] H. Steiner and C. R. Gerfen , “Cocaine‐Induced c‐Fos Messenger RNA Is Inversely Related to Dynorphin Expression in Striatum,” Journal of Neuroscience 13 (1993): 5066–5081, 10.1523/JNEUROSCI.13-12-05066.1993. 7504719 PMC6576421

[30] H. Steiner and C. R. Gerfen , “Role of Dynorphin and Enkephalin in the Regulation of Striatal Output Pathways and Behavior,” Experimental Brain Research 123 (1998): 60–76. 9835393 10.1007/s002210050545

[31] G. R. Valdez , D. M. Platt , J. K. Rowlett , D. Rüedi‐Bettschen , and R. D. Spealman , “Kappa Agonist‐Induced Reinstatement of Cocaine Seeking in Squirrel Monkeys: A Role for Opioid and Stress‐Related Mechanisms,” Journal of Pharmacology and Experimental Therapeutics 323 (2007): 525–533, 10.1124/jpet.107.125484. 17702903

[32] P. M. Beardsley , J. L. Howard , K. L. Shelton , and F. I. Carroll , “Differential Effects of the Novel Kappa Opioid Receptor Antagonist, JDTic, on Reinstatement of Cocaine‐Seeking Induced by Footshock Stressors vs Cocaine Primes and Its Antidepressant‐Like Effects in Rats,” Psychopharmacology 183 (2005): 118–126, 10.1007/s00213-005-0167-4. 16184376

[33] A. N. Carey , K. Borozny , J. V. Aldrich , and J. P. McLaughlin , “Reinstatement of Cocaine Place‐Conditioning Prevented by the Peptide Kappa‐Opioid Receptor Antagonist Arodyn,” European Journal of Pharmacology 569 (2007): 84–89, 10.1016/j.ejphar.2007.05.007. 17568579 PMC1994084

[34] V. A. Redila and C. Chavkin , “Stress‐Induced Reinstatement of Cocaine Seeking Is Mediated by the Kappa Opioid System,” Psychopharmacology 200 (2008): 59–70, 10.1007/s00213-008-1122-y. 18575850 PMC2680147

[35] R. Al‐Hasani , J. G. McCall , G. Shin , et al., “Distinct Subpopulations of Nucleus Accumbens Dynorphin Neurons Drive Aversion and Reward,” Neuron 87 (2015): 1063–1077, 10.1016/j.neuron.2015.08.019. 26335648 PMC4625385

[36] B. E. Pirino , M. B. Spodnick , A. T. Gargiulo , G. R. Curtis , J. R. Barson , and A. N. Karkhanis , “Kappa‐Opioid Receptor‐Dependent Changes in Dopamine and Anxiety‐Like or Approach‐Avoidance Behavior Occur Differentially Across the Nucleus Accumbens Shell Rostro‐Caudal Axis,” Neuropharmacology 181 (2020): 108341, 10.1016/j.neuropharm.2020.108341. 33011200 PMC8424943

[37] H. Pham , S. L. Seeley , and M. S. D'Souza , “Pharmacological Activation of Kappa Opioid Receptors in the Nucleus Accumbens Core and Ventral Tegmental Area Increases the Aversive Effects of Nicotine,” Behavioural Pharmacology 33, no. 4 (2022): 266–281, 10.1097/FBP.0000000000000675 35256559. 35256559

[38] K. Matsumura , A. Nicot , I. B. Choi , et al., “Endogenous Opioid System Modulates Conditioned Cocaine Reward in a Sex‐Dependent Manner,” Addiction Biology 28 (2023): e13328, 10.1111/adb.13328. 37753570 PMC11974355

[39] A. R. Soderman and E. M. Unterwald , “Cocaine Reward and Hyperactivity in the Rat: Sites of mu Opioid Receptor Modulation,” Neuroscience 154, no. 4 (2008): 1506–1516, 10.1016/j.neuroscience.2008.04.063 18550291. 18550291 PMC2585317

[40] R. A. Bevins and C. L. Cunningham , “Place Conditioning: A Methodological Analysis,” in Tasks and Techniques: A Sampling of Methodologies for the Investigation of Animal Learning, Behavior, and Cognition, ed. M. Anderson (Nova Science Publisher, 2006): 99–110.

[41] C. L. Cunningham , S. D. Dickinson , N. J. Grahame , D. M. Okorn , and C. S. McMullin , “Genetic Differences in Cocaine‐Induced Conditioned Place Preference in Mice Depend on Conditioning Trial Duration,” Psychopharmacology 146 (1999): 73–80. 10485967 10.1007/s002130051090

[42] C. R. Gerfen , R. Paletzki , and N. Heintz , “GENSAT BAC Cre‐Recombinase Driver Lines to Study the Functional Organization of Cerebral Cortical and Basal Ganglia Circuits,” Neuron 80, no. 6 (2013): 1368–1383, 10.1016/j.neuron.2013.10.016. 24360541 PMC3872013

[43] J. R. Goldsmith , E. Perez‐Chanona , P. N. Yadav , J. Whistler , B. Roth , and C. Jobin , “Intestinal Epithelial Cell–Derived μ‐Opioid Signaling Protects Against Ischemia Reperfusion Injury Through PI3K Signaling,” American Journal of Pathology 182, no. 3 (2013): 776–785, 10.1016/j.ajpath.2012.11.021 23291213. 23291213 PMC3589074

[44] L. K. Dobbs , A. R. Kaplan , J. C. Lemos , A. Matsui , M. Rubinstein , and V. A. Alvarez , “Dopamine Regulation of Lateral Inhibition Between Striatal Neurons Gates the Stimulant Actions of Cocaine,” Neuron 90 (2016): 1100–1113, 10.1016/j.neuron.2016.04.031. 27181061 PMC4891261

[45] J. C. Lemos , D. M. Friend , A. R. Kaplan , et al., “Enhanced GABA Transmission Drives Bradykinesia Following Loss of Dopamine D2 Receptor Signaling,” Neuron 90 (2016): 824–838, 10.1016/j.neuron.2016.04.040. 27196975 PMC4882167

[46] B. Schurmann and A. Zimmer , Endogenous Opioid Peptides in Drug Addiction (Rheinische Friedrich Wilhelms University, Bonn, 2010).

[47] M. D. Hayward , A. Schaich‐Borg , J. E. Pintar , and M. J. Low , “Differential Involvement of Endogenous Opioids in Sucrose Consumption and Food Reinforcement,” Pharmacology, Biochemistry, and Behavior 85, no. 3 (2006): 601–611, 10.1016/j.pbb.2006.10.015 17166571. 17166571 PMC1868438

[48] A. C. Shin , P. J. Pistell , C. B. Phifer , and H.‐R. Berthoud , “Reversible Suppression of Food Reward Behavior by Chronic mu‐Opioid Receptor Antagonism in the Nucleus Accumbens,” Neuroscience 170 (2010): 580–588, 10.1016/j.neuroscience.2010.07.017. 20654704 PMC2933316

[49] F. F. Caputi , L. Caffino , S. Candeletti , F. Fumagalli , and P. Romualdi , “Short‐Term Withdrawal From Repeated Exposure to Cocaine During Adolescence Modulates Dynorphin mRNA Levels and BDNF Signaling in the Rat Nucleus Accumbens,” Drug and Alcohol Dependence 197 (2019): 127–133, 10.1016/j.drugalcdep.2019.01.006. 30818133

[50] F. F. Caputi , M. Di Benedetto , D. Carretta , et al., “Dynorphin/KOP and Nociceptin/NOP Gene Expression and Epigenetic Changes by Cocaine in Rat Striatum and Nucleus Accumbens,” Progress in Neuro‐Psychopharmacology & Biological Psychiatry 49 (2014): 36–46, 10.1016/j.pnpbp.2013.10.016. 24184686

[51] J. B. Daunais and J. F. McGinty , “Acute and Chronic Cocaine Administration Differentially Alters Striatal Opioid and Nuclear Transcription Factor mRNAs,” Synapse 18 (1994): 35–45, 10.1002/syn.890180106. 7825122

[52] Y. L. Hurd and M. Herkenham , “Influence of a Single Injection of Cocaine, Amphetamine or GBR 12909 on mRNA Expression of Striatal Neuropeptides,” Brain Research. Molecular Brain Research 16 (1992): 97–104, 10.1016/0169-328x(92)90198-k. 1281257

[53] P. L. Smiley , M. Johnson , L. Bush , J. W. Gibb , and G. R. Hanson , “Effects of Cocaine on Extrapyramidal and Limbic Dynorphin Systems,” Journal of Pharmacology and Experimental Therapeutics 253 (1990): 938–943, 10.1016/S0022-3565(25)13241-2. 1972755

[54] R. Spangler , E. M. Unterwald , and M. J. Kreek , ““Binge” Cocaine Administration Induces a Sustained Increase of Prodynorphin mRNA in Rat Caudate‐Putamen,” Brain Research. Molecular Brain Research 19 (1993): 323–327, 10.1016/0169-328x(93)90133-a. 7694032

[55] Y. L. Hurd , E. E. Brown , J. M. Finlay , H. C. Fibiger , and C. R. Gerfen , “Cocaine Self‐Administration Differentially Alters mRNA Expression of Striatal Peptides,” Brain Research. Molecular Brain Research 13 (1992): 165–170, 10.1016/0169-328x(92)90058-j. 1374504

[56] C. L. Cunningham and D. Noble , “Methamphetamine‐Induced Conditioned Place Preference or Aversion Depending on Dose and Presence of Drug,” Annals of the New York Academy of Sciences 654 (1992): 431–433. 1632596 10.1111/j.1749-6632.1992.tb25989.x

[57] L. A. Parker , “Place Conditioning in a Three‐ or Four‐Choice Apparatus: Role of Stimulus Novelty in Drug‐Induced Place Conditioning,” Behavioral Neuroscience 106, no. 2 (1992): 294–306, 10.1037//0735-7044.106.2.294 1317184. 1317184

[58] R. M. Carelli and E. A. West , “When a Good Taste Turns Bad: Neural Mechanisms Underlying the Emergence of Negative Affect and Associated Natural Reward Devaluation by Cocaine,” Neuropharmacology 76 Pt B (2014): 360–369, 10.1016/j.neuropharm.2013.04.025. 23639430 PMC4160877

[59] D. S. Wheeler , M. A. Robble , E. M. Hebron , M. J. Dupont , A. L. Ebben , and R. A. Wheeler , “Drug Predictive Cues Activate Aversion‐Sensitive Striatal Neurons That Encode Drug Seeking,” Journal of Neuroscience 35 (2015): 7215–7225, 10.1523/JNEUROSCI.4823-14.2015. 25948270 PMC4420785

[60] R. A. Wheeler , R. C. Twining , J. L. Jones , J. M. Slater , P. S. Grigson , and R. M. Carelli , “Behavioral and Electrophysiological Indices of Negative Affect Predict Cocaine Self‐Administration,” Neuron 57 (2008): 774–785, 10.1016/j.neuron.2008.01.024. 18341996

[61] M. T. Bardo , J. S. Miller , and J. L. Neisewander , “Conditioned Place Preference With Morphine: The Effect of Extinction Training on the Reinforcing CR,” Pharmacology Biochemistry and Behavior 21 (1984): 545–549, 10.1016/S0091-3057(84)80037-4. 6504952

[62] R. Kumar , “Morphine Dependence in Rats: Secondary Reinforcement From Environmental Stimuli,” Psychopharmacologia 25 (1972): 332–338, 10.1007/BF00421972. 5065795

[63] J. E. Sherman , C. Pickman , A. Rice , J. C. Liebeskind , E. W. Holman , and J. E. Sherman , “Rewarding and Aversive Effects of Morphine: Temporal and Pharmacological Properties,” Pharmacology Biochemistry and Behavior 13, no. 4 (1980): 501–505, 10.1016/0091-3057(80)90271-3 7433482. 7433482

[64] C. Spyraki , A. Kazandjian , and D. Varonos , “Diazepam‐Induced Place Preference Conditioning: Appetitive and Antiaversive Properties,” Psychopharmacology 87 (1985): 225–232, 10.1007/BF00431813. 3931151

[65] J. Garcia and R. A. Koelling , “Relation of Cue to Consequence in Avoidance Learning,” Psychonomic Science 4 (1966): 123–124, 10.3758/BF03342209.

[66] A. G. Schindler , D. I. Messinger , J. S. Smith , et al., “Stress Produces Aversion and Potentiates Cocaine Reward by Releasing Endogenous Dynorphins in the Ventral Striatum to Locally Stimulate Serotonin Reuptake,” Journal of Neuroscience 32 (2012): 17582–17596, 10.1523/JNEUROSCI.3220-12.2012. 23223282 PMC3523715

[67] E. Chartoff , A. Sawyer , A. Rachlin , D. Potter , A. Pliakas , and W. A. Carlezon , “Blockade of Kappa Opioid Receptors Attenuates the Development of Depressive‐Like Behaviors Induced by Cocaine Withdrawal in Rats,” Neuropharmacology 62, no. 1 (2012): 167–176, 10.1016/j.neuropharm.2011.06.014. 21736885 PMC3195851

[68] N. Massaly , B. A. Copits , A. R. Wilson‐Poe , et al., “Pain‐Induced Negative Affect is Mediated via Recruitment of the Nucleus Accumbens Kappa Opioid System,” Neuron 102, no. 3 (2019): 564–573.e6, 10.1016/j.neuron.2019.02.029. 30878290 PMC6509001

[69] J. V. Aldrich , K. A. Patkar , and J. P. McLaughlin , “Zyklophin, a Systemically Active Selective Kappa Opioid Receptor Peptide Antagonist With Short Duration of Action,” Proceedings of the National Academy of Sciences of the United States of America 106 (2009): 18396–18401, 10.1073/pnas.0910180106. 19841255 PMC2775281

[70] M. Valenza , K. A. Windisch , E. R. Butelman , B. Reed , and M. J. Kreek , “Effects of Kappa Opioid Receptor Blockade by LY2444296 HCl, a Selective Short‐Acting Antagonist, During Chronic Extended Access Cocaine Self‐Administration and Re‐Exposure in rat,” Psychopharmacology 237 (2020): 1147–1160, 10.1007/s00213-019-05444-4. 31915862

