# Supplementary material for: Shifted Balance Between Ventral Striatal Prodynorphin and Proenkephalin Biases Development of Cocaine Place Avoidance
Source: Addict Biol. 2025 Jul 6;30(7):e70055. doi: 10.1111/adb.70055 (PMC12229727; doi:10.1111/adb.70055)
Supplement: Supplementary file 1 — Figure S1 Development of cocaine preference or avoidance is not determined by a preconditioning bias. (A–C) Data were stratified based on one standard deviation of the pretest mean, which created a neutral zone (indicated by dashed, grey lines; 40.28%–61.76%), flanked by subjects categorized as preferers (≥ 61.77%) and avoiders (≤ 40.27%). Conditioning scores at pretest and Post‐Test 2 are shown paired for each subject conditioned with 15 mg/kg (A), 20 mg/kg (B) or 25 mg/kg (C) cocaine. Data are expressed as individual values. Related to Figure 1. [file ADB-30-e70055-s001.pdf]

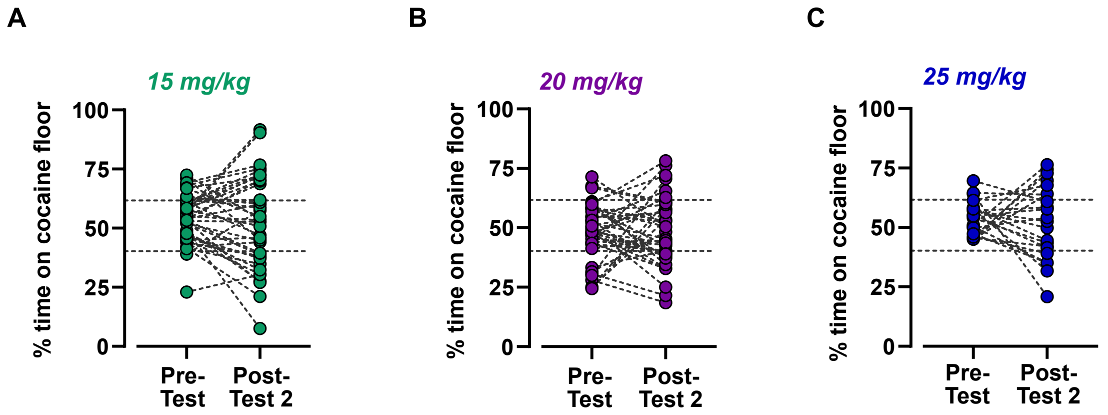

Supplemental Figure 1. Development of cocaine preference or avoidance is not determined by a pre-conditioning bias. (A-C) Data were stratified based on one standard deviation of the Pre-Test mean, which created a neutral zone (indicated by dashed, grey lines; 40.28 – 61.76%), flanked by subjects categorized as Preferers (≥ 61.77%) and Avoiders (≤ 40.27%). Conditioning scores at Pre-Test and Post-Test 2 are shown paired for each subject conditioned with 15 mg/kg (A), 20 mg/kg (B), or 25 mg/kg (C) cocaine. Data are expressed as individual values. Related to Figure 1.
